# GW5074 Increases Microglial Phagocytic Activities: Potential Therapeutic Direction for Alzheimer’s Disease

**DOI:** 10.3389/fncel.2022.894601

**Published:** 2022-05-23

**Authors:** Sarah M. Connor, Mamunur Rashid, Katie J. Ryan, Kruti Patel, Justin D. Boyd, Jennifer Smith, Wassim Elyaman, David A. Bennett, Elizabeth M. Bradshaw

**Affiliations:** ^1^Columbia University Irving Medical Center, New York, NY, United States; ^2^Ann Romney Center for Neurologic Diseases, Harvard Medical School, Brigham and Women’s Hospital, Boston, MA, United States; ^3^Laboratory for Drug Discovery in Neurodegeneration at the Harvard NeuroDiscovery Center, Harvard Medical School, Boston, MA, United States; ^4^The Institute of Chemistry and Cell Biology (ICCB)-Longwood Screening Facility, Harvard Medical School, Boston, MA, United States; ^5^Taub Institute for Research on Alzheimer’s Disease and the Aging Brain, New York, NY, United States; ^6^Alzheimer Disease Center, Rush University Medical Center, Chicago, IL, United States

**Keywords:** Alzheimer’s disease, microglia, GW5074, c-RAF, high content drug screening, TREM2, TYROBP

## Abstract

Microglia, the resident immune cells of the central nervous system (CNS), are responsible for maintaining homeostasis in the brain by clearing debris and are suggested to be inefficient in Alzheimer’s Disease (AD), a progressive neurodegenerative disorder for which there is no disease-modifying drug. Besides pathological approaches, unbiased evidence from genome-wide association studies (GWAS) and gene network analysis implicate genes expressed in microglia that reduce phagocytic ability as susceptibility genes for AD. Thus, a central feature toward AD therapy is to increase the microglial phagocytic activities while maintaining synaptic integrity. Here, we developed a robust unbiased high content screening assay to identify potential therapeutics which can reduce the amyloid-beta (Aβ1–42) load by increasing microglial uptake ability. Our screen identified the small-molecule GW5074, an inhibitor of c-RAF, a serine/threonine kinase, which significantly increased the Aβ1–42 clearance activities in human monocyte-derived microglia-like (MDMi) cells, a microglia culture model that recapitulates many genetic and phenotypic aspects of human microglia. Notably, GW5074 was previously reported to be neuroprotective for cerebellar granule cells and cortical neurons. We found that GW5074 significantly increased the expression of key AD-associated microglial molecules known to modulate phagocytosis: TYROBP, SIRPβ1, and TREM2. Our results demonstrated that GW5074 is a potential therapeutic for AD, by targeting microglia.

## Introduction

Alzheimer’s disease (AD) is a progressive disorder that destroys memory and cognition and is ultimately fatal, affecting millions of individuals with an immensely personal and social burden. It is considered among the top 10 killer diseases in the U.S. for people above 65 years with extremely limited options for drug treatments. Pathologically, AD is characterized by extracellular deposition of amyloid-beta (Aβ) plaques and intraneuronal hyperphosphorylation of the microtubule-associated protein, tau. Late-stage clinical trials using monoclonal antibodies targeting Aβ clearance failed to rescue cognition (Mullard, 2021), suggesting careful intervention for AD needs to be considered, where microglia phenotype itself is modified not just triggering Fc receptor (FcR)-induced phagocytosis. However, the FDA recently approved Aducanumab, a monoclonal antibody to target Aβ, using an accelerated approval scheme (FDA, 2021). While this approval seems promising, it has been highly criticized by the scientific community, and the clinical effectiveness will be clearer after the post-approval trials. Thus, the real effectiveness of the approved drug is currently unclear and pending (Barenholtz Levy, 2021). Historically, the scientific community had been focused mainly on neurons to understand the etiology of AD with less attention given to the supporting cells like microglia and astrocytes. This changed with genome-wide association studies (GWAS), which implicate innate immune cells in the AD brain. GWAS and transcriptomic studies repeatedly identified many genetic loci and gene networks that involve myeloid cells and are involved in microglial phagocytosis, for example, CD33, TREM2, TYROBP, ABCA7, SIRPβ1, CR1, BIN1, and PICALM (Lambert et al., 2013; Haure-Mirande et al., 2019; Bis et al., 2020).

It is very clear from human genetics that a microglia-specific intervention would be a more strategic way to treat sporadic or late-onset AD (LOAD). Thus, to specifically target microglia, we performed a high content drug screen with a microglial culture model, monocyte-derived microglia-like (MDMi) cells. Previously, we showed that MDMi are genetically and phenotypically similar to human microglia making this an ideal model for drug screens with different genetic backgrounds accounted for Ryan et al. (2017). Utilizing drug repurposing to identify potential therapeutics that target microglial phagocytosis for AD treatment, we performed a drug screen using the Library of Pharmacologically Active Compounds (LOPAC1280), a collection of 1,280 chemicals with known biological function. Our screen identified several hits which increase the uptake of Aβ1–42 by MDMi in the presence of the drugs. Among the hits, GW5074 [IUPAC name: 3-(3,5-Dibromo-4-hydroxybenzyliden)-5-iodo-1,3-dihydroindol-2-one], a potent inhibitor of the c-RAF kinase, significantly increased the Aβ1–42 uptake ability of the MDMi.

Protein kinases are the best-characterized molecules to transmit receptor-mediated extracellular signals to the nucleus, ultimately known to regulate proliferation, differentiation, and apoptosis. In this regard, the so-called RAF/MAP kinase pathway is extensively studied, where sequential activation of RAF, MEK, and ERK kinases occurs. The fine-tuning of these kinases plays a central role in cell signaling and normal cellular function (Robinson and Cobb, 1997; Pearson et al., 2001), while dysregulation is implicated in many forms of cancer (Dhillon et al., 2007). c-RAF inhibition was previously reported to be neuroprotective in neuronal cultures and in an *in vivo* mouse model of Huntington’s disease (Chin et al., 2004; Burgess and Echeverria, 2010). For example, GW5074 prevents low potassium-induced apoptosis or 1-methyl-4-phenylpyridinium (MPP+) and methylmercury mediated toxicity of cerebellar granule neurons. Similarly, in a Huntington’s disease mouse model GW5074 was reported to prevent neurodegeneration and improve behavior, making it a potential therapeutic candidate for neurodegeneration (Chin et al., 2004). While the prior studies focused on neurons, the effect of GW5074 is unknown in the context of microglia or phagocytosis in general. Our screening identifies GW5074 as a significant modifier of phagocytosis, which significantly increases the uptake of Aβ1–42 in our human microglia-like culture model. We additionally identified that GW5074 increases the expression of major phagocytic molecules that are associated with LOAD. Thus, this study provides a novel direction toward a microglial-directed AD therapy.

## Materials and Methods

### Study Subjects

Informed consent was obtained from all human subjects. All blood draws, experiments and data analysis were done in compliance with protocols approved by the Partners, RUSH or Columbia Human Research Committee.

### Rush Memory and Aging Project

The details about the Rush Memory and Aging Project is published elsewhere (Bennett et al., 2018). For this project, PBMCs from 65 persons without dementia and seven with a diagnosis of clinical AD (cAD) were used. The participants were recruited unbiasedly without knowing their clinical status. That’s why there is a disparity between the number of healthy subjects and AD subjects. Both sexes were included in the experiments. The average ages of those without dementia and cAD were 86 and 89 years, respectively. Among the non-demented subjects, there were 13 males and 52 females. For the cAD group, there were six females and one male. MAP was approved by an Institutional Review Board of Rush University Medical Center. All participants signed an informed consent, Anatomical Gift Act, and repository consent allowing their data to be repurposed. MAP resources can be requested at https://www.radc.rush.edu.

### Crimson Core

Blood samples were anonymously collected from Partners’ Crimson Biospecimen Repository Core. The crimson core (CC) offers IRB-compliant access to samples and generates an anonymous sample identifier so that no original identifiers (laboratory accession number, medical record number, etc.) remain associated with the sample.

### The PhenoGenetic Project

Peripheral venous blood was obtained from healthy volunteers from the Brigham and Women’s Hospital PGP. The PhenoGenetic project (PGP) was launched as a living biobank that provides a source of fresh and frozen biological samples derived from peripheral blood, urine, and saliva of genotyped human subjects. 1,753 healthy subjects > 18 years old have been recruited from the general population of Boston. Subjects are free of chronic inflammatory, infectious, and metabolic diseases and are of diverse ethnicities (29% are non-Caucasian) and are 62.7% women. The median age is 24.

### New York Blood Center

The New York Blood Center (NYBC) provided IRB-compliant access to blood samples of de-identified individuals. The donors range in age from 17 to 75 years of age and are both male and female. All validation studies were performed using PGP or NYBC subjects.

### Genotyping

For the Crimson Core subjects, genomic DNA (gDNA) was purified from peripheral blood mononuclear cells (PBMCs) using the Gentra Puregene Blood Kit (Qiagen). DNA quantification was performed using the NanoDrop ND-1000 spectrophotometer (NanoDrop Technologies). The genotype for CD33 was determined using Custom Taqman SNP Genotyping Assay (Thermo Fisher Scientific, Assay ID AHLJYS8, Waltham, MA, United States) on the QuantStudio 7 Flex Real-Time PCR System. Approximately, 20 ng of gDNA was used in each reaction. High-throughput screening was performed with samples from individuals with the CD33 AD-risk genotype.

### Collection of Human Peripheral Blood Mononuclear Cells

PBMCs were separated by Lymphoprep gradient centrifugation (StemCell Technologies). PBMCs were frozen at a concentration of 1–3 × 10^7^ cells ml^–1^ in 10% DMSO (Sigma-Aldrich)/90% fetal bovine serum (vol/vol, Corning). After thawing, PBMCs were washed in 10 ml of phosphate-buffered saline according to a previously published protocol (Ryan et al., 2017).

### Monocyte to Microglia-Like Cell Culture

MDMi cultures were generated as previously described (Ryan et al., 2017). Briefly, monocytes were positively selected from whole PBMCs using anti-CD14 + microbeads (Miltenyi Biotech, Auburn, CA, United States). Cells were seeded and stimulated with RPMI-1640 Glutamax with 1% penicillin/streptomycin, 1% Fungizone and a cytokine cocktail that consisted of 0.01 μg/ml NGF-β (Biolegend Inc., San Diego, CA, United States), 0.01 μg/ml GM-CSF (R&D Systems, Minneapolis, MN, United States), 0.01 μg/ml M-CSF (R&D Systems, Minneapolis, MN, United States), 0.1 μg/ml IL-34 (R&D Systems, Minneapolis, MN, United States), and 0.1 μg/ml CCL2 (Biolegend Inc., San Diego, CA, United States). Cells were seeded at 1.7 × 10^4^ cells/60 μl in 384-well plates (Corning) and cultured for 9 or 10 days at standard humidified culture conditions (37°C, 5% CO_2_).

### Compound Screen

The screen was performed with 1,280 bioactive compounds from The Library of Pharmacologically Active Compounds (LOPAC) purchased from Sigma-Aldrich (St. Louis, MO). This is a collection of marketed drugs and pharmaceutically relevant structures annotated with biological activities. On day 9, human MDMi were incubated at 37°C with each of the compounds with a final concentration of 33 μM for 24 h. On day 10, cells were incubated with FITC-labeled dextran (Fluorescein, 40,000 MW, Anionic, Lysine Fixable, Thermo Fisher Scientific D1845, Waltham, MA, United States) for 2 h at 37°C. Cells were labeled with live dead red cell stain (L34971, Thermo Fisher Scientific, Fremont, CA, United States), fixed with 4% PFA, and labeled with DAPI. Cells were imaged on the ImageXpress Micro Confocal (Molecular devices, San Jose, CA, United States). Screens were performed in duplicate. A compound was considered a “hit” and selected for further confirmation if its dextran fluorescence mean cell intensity was greater than the mean plus the standard deviation of the negative control. Any compounds that induced three times below the standard deviation of the negative control wells were considered to be cytotoxic and not moved forward.

### Validation of Hits With Aβ1–42

For primary validation, MDMi were seeded at 1.5 × 10^5^ cells/200 μl in a 96 well plate. MDMi were treated with one of the six hits (at 33 μM) for 24 h at 37°C. Cells were incubated with 1.5 μg/ml of HyLite Fluor-488 conjugated Aβ 1–42 (AnaSpec, Cat# AS-60479-01, Fremont, CA, United States) for 2 h at 37°C, labeled with live dead red cell stain (L34971, Thermo Fisher Scientific, Waltham, MA, United States), fixed with 4% PFA and labeled with DAPI. Cells were imaged on the ImageXpress Micro Confocal (Molecular devices, San Jose, CA, United States). FITC mean cell intensity was quantified.

### Flow Cytometry Analysis

Monocytes were seeded at 1.5 × 10^5^/200 μl and cultured for 9 days with RPMI with MDMi cytokine cocktail on temperature-sensitive plates (Thermo Fisher Scientific, Cat# 03150018, Waltham, MA, United States). On day 9, cells were transferred to a 96 well polypropylene plate and incubated with 100 μM of GW5074 at 37°C for 24 h. Cells were stained on ice with TREM2-APC (R&D Systems, Cat# FAB17291A, Minneapolis, MN, United States) for 30 min away from light. Cells were stained for LIVE/DEAD™ Fixable Blue Dead Cell stain kit (Thermo Fisher Scientific, Cat# L23105, Waltham, MA, United States) and fixed in 4% PFA. Flow cytometry data were acquired by using the BD LSR II Flow Cytometer (BD Biosciences) at the Flow Cytometry Core of the Columbia Center for Translational Immunology (CCTI). Data were post analyzed using FlowJo (BD Bioscience).

### Immunocytochemistry

For immunocytochemistry, on day 10, MDMi media was removed and washed with 200 μl staining buffer (5% FBS in PBS) followed by 100 μl LIVE/DEAD™ Fixable Red Dead Cell Stain Kit (Thermo Fisher Scientific, cat # L34971, Waltham, MA, United States) for 30 min on ice and covered from light. After washing with staining buffer, cells were fixed with 4% PFA for 15 min followed by washing with PBS. Cells were then blocked with 3% BSA for 30 min and covered from light. Then they were stained with antibody in 100 μl of 3% BSA for 30 min on ice and covered from light followed by washing with staining buffer, followed by a secondary antibody, when necessary. Cells were then resuspended in 200 μl of staining buffer. Imaging was performed on the Celigo Imaging Cytometer (Nexcelom Bioscience, Lawrence, MA, United States). Analysis was performed using the software built-in to the Celigo instrument. The images were acquired as 8-bit images, and cells were gated only on live cells. The mean intensity (in a 255 scale for 8-bit image) of the images were collected. The following antibodies were used: TYROBP (R&D Systems, Cat# MAB5240, Minneapolis, MN, United States) and SIRPβ1 (Biolegend, Cat# 337304, San Diego, CA, United States). SIRPβ1 was conjugated to APC. The secondary antibody for TYROBP was conjugated with Alexa Fluor 488 (Thermo Fisher Scientific, Cat# R37114, Waltham, MA, United States).

## Lentivirus Mediated shRNA Knockdown in Monocyte-Derived Microglia-Like Cells

### Preparation of shRNA Lentiviral Particle

On day 1, 293T cells were transfected using Lipofectamine 2000 (Thermo Fisher Scientific, Waltham, MA, United States) with packaging and envelope plasmids (Vpx cDNA and pHEF-VSVG). On day 2, 293T culture media was replaced with RPMI-1640 Glutamax (Invitrogen, Waltham, MA, United States) containing 1% fungizone (Amphotericin B) and 1% penicillin/streptomycin. After 48 h, lentiviruses containing the Vpx particles were harvested, centrifuged for 5 min at 400 g and the supernatant collected. The supernatant was filtered using a 0.45-μm syringe filter (EMD Millipore, Burlington, MA, United States). Lentiviral particles containing targeted shRNA for each gene were obtained from the Broad Institute- TYROBP (Construct 1: TRCN0000423493, target sequence: TATTACAAATGAGCCCGAATC; Construct 2: TRCN0000037 830, target sequence: TCAACACACAGAGGCCGTATT; and construct 3: TRCN0000419502, target sequence: GATACCTGGA TCCAGCCATTC) and c-RAF (construct 1: TRCN0000001068, target sequence: GAGACATGAAATCCAACAATA construct 2: TRCN0000001065, target sequence: GCTTCCTTATTCTCAC ATCAA; and construct 3: TRCN0000197115, target sequence: GCTCAGGGAATGGACTATTTG).

### Lentivirus Mediated Knockdown of Monocyte-Derived Microglia-Like

For the transduction of MDMi cells, on day 3 of differentiation, 100 μl of Vpx-VLP was added to the media, followed by 10 μl TRC virus-containing shRNAs or empty pLKO.1 control (Sigma). On day 7, puromycin (Life Technologies, Carlsbad, CA, United States) at a concentration of 3 μg/ml was added to eliminate non-transduced cells. On day 10, MDMi were lysed for RNA isolation. A > 80% knockdown of RNA (by qPCR) was considered optimal for the experiment.

### Statistical Analysis

Statistical analyses were performed using GraphPad Prism (GraphPad Software, San Diego, CA, United States). At least three (*n* = 3) subjects were used for any given test. Data were presented as mean ± SEM. For group-wise comparison, one-way-ANOVA was used with Dunnett’s multiple comparisons test. Student’s *t*-test was performed for comparison between groups. A *p*-value less than 0.05 is considered significant.

## Results

### Monocyte-Derived Microglia-Like From Alzheimer’s Disease Patients Are Less Effective in Aβ1–42 Uptake

**FIGURE 1:**
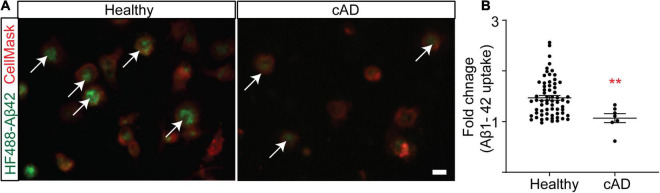
Clinical AD (cAD) subjects are ineffective at phagocytosis compared to healthy subjects in RUSH Universities’ Memory and Aging Project (MAP) (Bennett et al., 2012; Bennett et al., 2018). Monocytes were seeded into a 384 well plate and differentiated to MDMi. **(A)** A representative image of MDMi incubated with HyLite Fluor-488 conjugated Aβ 1–42 for 2 h. CellMask was used to label the plasma membrane. Arrow indicates internalized Aβ in the healthy subjects. **(B)** Quantification of the mean fluorescence intensity of Aβ, represented as fold change. *N* = 65 for healthy groups and *N* = 7 for cAD group. Data were analyzed using non-parametric Mann Whitney test. Scale bar, 10 μm. **p < 0.01. cAD, clinical Alzheimer’s disease. Each dot represents individual subjects.

Genetic studies suggest that genes of myeloid origin such as TREM2 and CD33 are implicated in AD, linking the innate immune component to the pathophysiology and therapeutic direction of this disorder (Harold et al., 2009; Lambert et al., 2009; Seshadri et al., 2010; Hollingworth et al., 2011; Naj et al., 2011; Guerreiro et al., 2013; Jin et al., 2014). The rate of Aβ1–42 clearance is impaired in the AD brain (Wildsmith et al., 2013), suggesting an imbalance between Aβ production and clearance. While the innate immune system has many roles in the central nervous system (CNS), CD33 and TREM2 have been linked to impaired phagocytosis of Aβ1–42 in AD. Indeed, monocytes and monocyte-derived macrophages from AD patients were found to be ineffective in Aβ phagocytosis (Fiala et al., 2005; Avagyan et al., 2009; Zaghi et al., 2009). Similarly, a more recent study based on stem cell-derived microglia-like cells found that TREM2 deficient microglia are less effective at Aβ clearance (Claes et al., 2019). SIRPβ1, also a TYROBP signaling receptor, was additionally found to be important for amyloid and neural debris clearance by microglia (Gaikwad et al., 2009). Here we polarized primary human monocytes in a CNS milieu cytokine cocktail to create MDMi, which we have previously found to up-regulate a number of the genes recently identified as part of the microglia signature (Ryan et al., 2017). We hypothesized that MDMi from subjects with a diagnosis of clinical AD (cAD) have impaired phagocytosis. To test the hypothesis, we generated MDMi from the Memory and Aging Project (MAP) cohort which is a longitudinal clinical-pathologic study of aging and AD. We used fluorescently labeled Aβ1–42 to quantify phagocytosis in MDMi and found that phagocytosis was significantly reduced, by 27%, in cAD patients as compared to healthy subjects (Figures 1A,B; Healthy control = 1.47 ± 0.05%, cAD = 1.07 ± 0.09%, *p* = 0.0076). This result correlates with the prior observations of a myeloid cell functional defect in AD.

### Library of Pharmacologically Active Compounds Screening for Microglial Phagocytic Modulators

**FIGURE 2:**
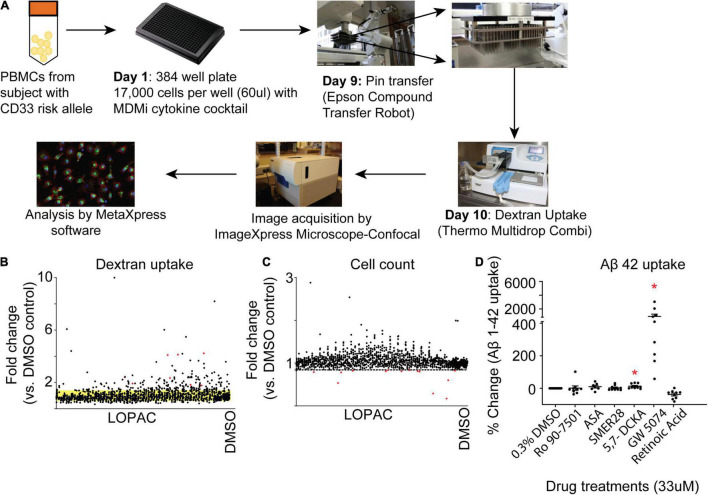
Identification of hit compounds. **(A)** Schematic of the workflow of the screening process. Monocytes were seeded into a 384 Well Flat Clear Bottom Black Polystyrene Microplate and differentiated to MDMi. On day 9, each well was incubated with a single compound (except for DMSO) for 24 h. The experiment was performed in duplicate. Scatterplot graph is of the first-step screening. **(B)** Fold change of drug screen compounds compared with the DMSO control were plotted according to the FITC-labeled dextran uptake. The standard deviation of the DMSO control groups is indicated by the yellow band. **(C)** Fold change of drug screen compounds compared with DMSO control were plotted according to cell count (DAPI). The dotted line indicates three standard deviations below the mean cell count of the negative control. Any drugs below this line are cytotoxic and were excluded from the screen. **(D)** Hit validation in MDMi with Aβ1–42. Monocytes were seeded into a 384 well plate and differentiated to MDMi. Each well was treated with a single hit compound (33 μM) for 24 h. Cells were incubated with HyLite Fluor conjugated Aβ1–42 for 2 h, labeled with CellMask and imaged using the IXM-C. Data expressed as mean percent change compared to DMSO control. Data were analyzed using Student’s *t*-test. *p < 0.05. Each dot represents individual subjects.

To identify a pharmaceutical target that modulates the innate immune system and increases phagocytic activities of myeloid cells, we screened for different chemical agents using The Library of Pharmacologically Active Compounds (LOPAC1280) with the MDMi culture model. LOPAC1280 is a biologically annotated collection of 1,280 pharmacologically active receptor ligands, inhibitors, and approved drugs that have a broad range of signaling and drug target pathways (Sigma-Aldrich). After 24 h of incubation with the LOPAC compounds, we initially used FITC-conjugated dextran to measure the dextran uptake by MDMi cultures generated from a young healthy individual homozygous for the CD33 AD risk allele (rs3865444^CC^), which is known to have reduced uptake ability compared to the CD33 AD protective allele (rs3865444^AA^) (Bradshaw et al., 2013). The dextran uptake assay serves as a surrogate assay of phagocytosis/endocytosis (Figure 2A). Detailed data from the screen is provided as Supplementary Data. To determine the most relevant molecules, a compound was considered a “hit” and selected for further confirmation if its dextran mean fluorescence intensity was four times greater than the mean standard deviation of the negative control (Figure 2B). For the negative control 0.3% DMSO was considered optimum for cell survival and signal intensity for the uptake assay (Supplementary Figure 1). This screen was performed based on cell count (quantified by DAPI) as a measure for cell viability. We set a “threshold” as 3 standard deviations below the mean of the negative control (DMSO). Any drugs that fell below this threshold were considered too cytotoxic and were excluded from our positive hit list (Figure 2C). Based on these criteria 81 molecules passed the threshold. From the 81 hits we proceeded with the top six hits based on commercial availability and their known targets. These six molecules include Ro 90–7,501, a known inhibitor of Aβ1–42 fibril formation; acetylsalicylic acid (ASA), a Cox-1/2 inhibitor; SMER28, a modulator of mammalian autophagy; 5,7-Dichlorokynurenic Acid, an NMDA receptors antagonist; GW5074, c-RAF kinase inhibitor; Retinoic Acid, a ligand for both retinoid X receptor (RXR) and retinoic acid receptor (RAR) (Supplementary Table 1). Due to limitations, the screen for plate 3,260 was performed and analyzed separate to the rest of the screen, and therefore is not included in the final list of hits (except for acetylsalicylic acid which was a hit chose for further validation).

Next, we validated these six molecules with the more biologically relevant compound Aβ1–42 which is conjugated with HiLyte™ Fluor 488 in additional MDMi samples. Upon validation with Aβ1–42, only two molecules significantly increased uptake, 5,7-Dichlorokynurenic acid and GW5074 (Figure 2D). Among all these potential phagocytic modulators, GW5074 is the top one which significantly increases Aβ1–42 uptake by more than 900% compared to the DMSO treated controlled group (Figure 2D; GW5074 = 904 ± 340% change vs. DMSO control), leading us to focus additional experiments on the drug GW5074.

### GW5074 Is a Potential Phagocytic Modulator of Microglia

**FIGURE 3:**
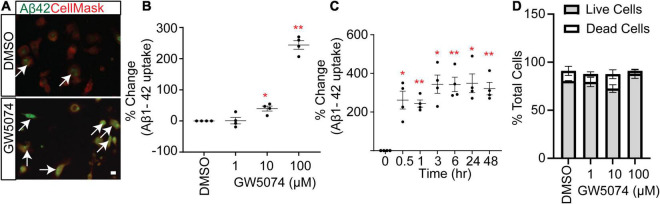
GW5074 optimization in MDMi with amyloid-β 1–42. **(A)** A representative image of MDMi treated with either DMSO or GW5074 and incubated with HyLite Fluor-488 conjugated Aβ1–42 for 2 h. CellMask was used to label the plasma membrane. Arrows indicate increased internalized Aβ in the GW5074 treated MDMi. **(B)** MDMi were treated with different concentrations of the drug for 24 h and subsequently incubated with HyLite Fluor-488 conjugated Aβ1–42 for 2 h. Cells were imaged on the Celigo Imaging Cytometer (Nexcelom Bioscience) and measured for live cell Aβ1–42 signal intensity. **(C)** Cells were incubated with 100.μM GW5074 for different time points and the intensity of Aβ1–42 was measured. **(D)** Different doses of GW5074 plotted against cell viability using Live/Dead cell staining kit. No obvious cell death was observed. Data expressed as mean percent change compared to DMSO control. Data were analyzed using Student’s *t*-test. *p < 0.05, **p < 0.01. Scale bar, 10 μm. Each dot represents individual subjects.

Our screen identified GW5074, a potent inhibitor of the c-RAF kinase, as a potential small molecule that significantly increased the uptake of Aβ1–42 by MDMi cultures. While the RAF kinase family has been studied extensively in the cancer field, it is less explored in the context of neuroimmunology. Interestingly GW5074 was previously reported as neuroprotective in a murine model of Huntington’s disease and it can cross the blood-brain barrier (Chin et al., 2004). Imaging shows the Aβ1–42 signal in the GW5074 treated MDMi compared to the 0.3% DMSO vehicle-treated MDMi is increased (Figure 3A). We performed a dose-response of GW5074 at different concentrations and found that Aβ1–42 uptake increases with the concentration of the drug (Figure 3B; % change for 1 μM = 0.85 ± 10.39%; for 10 μM = 39.7 ± 8.05%; and for 100 μM = 243.8 ± 13.9%). Additionally, we measured the mean fluorescence intensity of Aβ1–42 uptake at different time points of GW5074 incubation with MDMi, ranging from 30 min to 48 h (Figure 3C; % change for 0.5 h = 261.7 ± 46.08%; for 1 h = 243.7 ± 18.85%; for 3 h = 343.6 ± 47.38%; for 6 h = 342.6 ± 37.74; for 24 h = 348.6 ± 48.34%; and for 48 h = 321.2 ± 32.16%), and found that the increase in uptake occurred as early as 30 min. This suggests that GW5074 is a potential modulator of MDMi phagocytic functions and may provide additional direction for AD therapeutics development. The dose-response curve shows that GW5074 does not have potential cell viability effects when tested using 1, 10, and 100 μM (Figure 3D).

### GW5074 Increases Genetically Associated Alzheimer’s Disease Microglial Phagocytic Proteins

**FIGURE 4:**
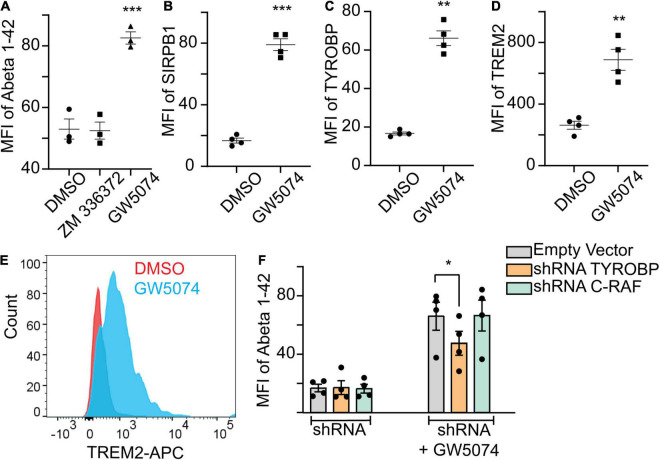
GW5074 upregulates SIRPβ1, TYROBP, and TREM2 protein expression. Monocytes were seeded into a 96 well plate and differentiated to MDMi for 10 days. **(A)** 100 μM ZM336372 treatment does not increase the Abeta1–42 uptake suggesting the phagocytosis may be mediated through a c-RAF independent pathway. GW5074 (100 μM) increases the uptake significantly. **(B,C)** Cells were treated with 100 μM GW5074 for 24 h. Cells were stained intracellularly for TYROBP and extracellularly for SIRPβ1 followed by imaging on the Celigo Imaging Cytometer. **(D)** For TREM2 cells were stained extracellularly and quantified using flow cytometry. **(E)** Representative histogram of TREM2 staining via flow cytometry. **(F)** GW5074 had reduced Abeta1–42 uptake when TYROBP is knocked down (using construct 3; Supplementary Figure 2) by using lentivirus mediated targeted shRNA, suggesting GW5074 is working through TYROBP signaling to facilitate Aβ1–42 uptake. The knockdown of c-RAF (construct 3) does not have any effect on Aβ1–42 uptake in the presence of GW5074, suggesting the uptake mechanism is not dependent on the c-RAF pathway. Data is expressed as mean percent change compared to DMSO control. Data were analyzed using Student’s *t*-test. *p < 0.05, **p < 0.01, ***p < 0.001. Each dot represents individual subjects.

GW5074 is a known c-RAF inhibitor. Therefore, we examined the ability of ZM336372, another potent c-RAF inhibitor, to increase Aβ1–42 uptake by MDMi. We found no increase of Aβ1–42 uptake with 100 μM ZM336372 (Figure 4A), suggesting a non-canonical mechanism for GW5074. GWAS studies and gene network analysis identified many microglial or immune genes to be associated with AD and phagocytosis. We found that GW5074 significantly increased the uptake of Aβ1–42, thus we hypothesized that GW5074 upregulates the molecules that are genetically linked to microglial phagocytosis of Aβ1–42. TYROBP (AKA DAP12), SIRPβ1 (Gaikwad et al., 2009) and TREM2 (Takahashi et al., 2005; Kleinberger et al., 2014; Kim et al., 2017) are important proteins that are known to regulate phagocytosis (Haure-Mirande et al., 2019). We performed immunostaining for these proteins in the MDMi culture upon treatment with GW5074. GW5074 treatment significantly increases the protein expression of all three of these molecules compared with the DMSO control group (Figures 4B–E). Previous studies showed that TREM2/SIRPβ1/DAP12 is one of the major axes for microglia phagocytosis and homeostasis of the brain (Gaikwad et al., 2009; Haure-Mirande et al., 2019), thus suggesting GW5074 preferentially activates this phagocytic machinery. To help determine the mechanism of action, shRNA was used to reduce the expression of c-RAF, the known target of GW5074 as well as TYROBP, the key signaling molecule in the TREM2/SIRPβ1/TYROBP axis. Reduction of c-RAF had no effect on MDMi uptake ability, while reduction of TYROBP reduced the uptake of Aβ1–42 compared to the vector control, suggesting a novel mechanism for GW5074 (Figure 4F and Supplementary Figure 2).

## Discussion

Microglial dysfunction, particularly altered phagocytosis, is thought to play a vital role in the progression of AD (Lee and Landreth, 2010); thus, therapeutic modulation of microglial function is of great interest yet there are no available therapies to date. Both human and mouse microglia are a challenge to work with *in vitro* as they both rapidly de-differentiate to a generic myeloid phenotype (Gosselin et al., 2017), and it is difficult to obtain the numbers required for drug screening. Induced pluripotent stem cell-derived microglia have become a great option, however, they are limited in the number of individuals who can be examined. In the current study, we used high content drug screening paired with a monocyte-derived microglia-like model, where primary cells are used to create microglia-like cells from an accessible tissue, to identify potential therapeutics toward improving microglial phagocytosis via upregulation of key known microglial phagocytic proteins.

The identification of the c-RAF kinase inhibitor, GW5074, as the potential modulator for microglial phagocytosis, is novel and surprising considering its identification and research over the last four decades. c-RAF is intensely studied in many cellular systems, especially in the context of cancer biology, but it was understudied in microglia, partly due to the difficulty of accessing these cells and early embryonic lethality of c-RAF knockout mice prior to the proper microglial invasion to the CNS (Wojnowski et al., 1998). The target of GW5074 is well defined and several studies have confirmed that it is a selective inhibitor of the c-RAF kinase. Targeted knockout studies identified that c-RAF can use its kinase-independent activities to induce apoptosis and migration via physically interacting and inhibiting other kinases such as MST2 (O’NEILL et al., 2004), ASK1 (Yamaguchi et al., 2004), and Rok-α (Ehrenreiter et al., 2005; Varga et al., 2017). Whether all three family member kinases can compensate for each other is not fully resolved and is partly obscured by their tissue-specific differential expression pattern. For example, the activation of ERK is reported in fibroblasts lacking either c-RAF or a-RAF (Mercer et al., 2002), while a 60% reduction in ERK activation is reported in fibroblasts lacking b-RAF (Mikula et al., 2001; Galabova-Kovacs et al., 2006).

In the context of degenerating neurons, the aberrant c-RAF/MEK/ERK pathway is implicated in the AD brain and in an animal model of Huntington’s disease (Echeverria et al., 2004; Mei et al., 2006). In the AD postmortem brain, the amount of phosphorylated c-RAF at S259 and/or S338 residues is significantly increased (Mei et al., 2006), suggesting altered activation of this kinase in the progression of AD. GW5074 is a potent inhibitor of c-RAF kinase and thus its effect on neuroprotection is implicated in several studies. Importantly, in cortical neurons cultured with Aβ1–42, GW5074 was shown to be protective via suppression of NF-κB signaling (Echeverria et al., 2008). In contrast, it was also reported that the neuroprotective property of the drug is mediated through the activation of b-RAF which inhibits transcription factor 3 (ATF3) activation (Chen et al., 2008). The status of b-RAF in our study is unknown, however, as suggested by the knockout studies, there could be a compensatory mechanism to activate the ERK pathway by other RAF kinases. These neuroprotective functions of GW5074 make it an attractive molecule for neurodegenerative diseases. However, the true/preferential specificity of Aβ1–42 is not fully resolved in this study since the screening also shows that dextran uptake is also increased with GW5074. This raises the question of whether this drug could introduce any detrimental effect on the neurons such as synaptic stripping is currently unknown from this study.

To our knowledge the finding that GW5074 increases microglial uptake activities is novel. Our results on microglia-like cells, and prior studies on neurons, point toward the potential of this drug or a derivative in the treatment of neurodegenerative disorders. Although GW5074 is a potent inhibitor of the c-RAF kinase, the phagocytic activities we observed in our experiment appear to be independent of it. Indeed, a pan c-RAF kinase inhibitor, ZM336372, did not increase uptake ability. Also, shRNA reduction of c-RAF expression did not influence the ability of GW5074 to increase the uptake of Aβ1–42 (Figure 4F). The current study does not exclude the possibility that GW5074 is inhibiting the MAP kinase pathway in MDMi but rather that the increased Aβ1–42 uptake ability induced byGW5074 is independent of ERK/MAP kinase pathway. Therefore, we hypothesize that GW5074 may be influencing the TREM2/SIRPβ1/TYROBP axis, which has been implicated in various studies to the phagocytosis of Aβ1–42. Indeed, we found an increase in protein expression of all three molecules with the treatment of GW5074, and shRNA reduction of TYROBP lead to reduced uptake compared to an empty vector. While this study is the first step toward a drug screening platform utilizing MDMi from patients, there are some limitations of the current study. The effect of GW5074 directly on MDMi derived from AD patients is unknown from our study. It would be interesting to examine GW5074 with AD patients derived MDMi and in iPSC derived neuron-MDMi coculture. In this study, we have used monomeric forms of Aβ1–42. How GW5074 responds to other forms of Aβ1–42 such as oligomeric or fibrillar is currently unknown. It is known that oligomers and fibrillar forms of Abeta have differential effects on neuronal health (Dahlgren et al., 2002; Walsh et al., 2002). Thus, it would be interesting to see if the GW5074 clears the oligomers/fibrillar form of Aβ1–42 without producing significant proinflammatory cytokines.

Genetic evidence has shown that variants in TREM2, which is exclusively expressed by innate immune cells, are associated with AD development (Guerreiro et al., 2013; Jonsson et al., 2013). Functional studies demonstrate that knocking down microglial TREM2 decreases their phagocytic activities, while overexpression showed the opposite effect (Takahashi et al., 2005). Similarly, expression of the AD variants of TREM2 in the microglia cell line BV2 showed impaired phagocytosis (Kleinberger et al., 2014). TREM2 is currently being targeted in clinical trials for AD, in that patients are being treated with agonistic antibodies in an effort to increase TREM2 signaling (Chatila and Bradshaw, 2021). Membrane-bound TREM2 signals via its association with TYROBP which contains an immunoreceptor tyrosine-based activation motif (ITAM) in its cytoplasmic domain. TYROBP interacts with spleen tyrosine kinase (Syk) via these ITAM domains to activate the cell (Peng et al., 2010). Interestingly, SIRPβ1 is also known to interact with and signal via TYROBP and has also been shown to be important for microglial phagocytosis (Gaikwad et al., 2009). Thus, GW5074 helps increase the TREM2/SIRPβ1/TYROBP signaling complex which we suggest increases the phagocytic activities and Aβ1–42 engulfment. In this study, we did not directly look into the downstream molecules of TREM2/TYROBP signaling. Specifically, as stated above, the activation status of Syk would be interesting to follow up. Similarly, the downstream effects of c-RAF are not studied and unknown to this study. Thus, a converging signaling axis utilizing c-RAF and TYROBP can be further studied. Indeed, others have shown that the activation of the Syk/PI3K/AKT/PLCγ pathway is required for TREM2 dependent phagocytosis (Yao et al., 2019). Thus, an emerging model arises from this study requiring examination of the downstream signaling of both of the pathways.

As microglia have become a central theme in our current understanding of AD progression, and this is backed up by genetic evidence, we show that the Aβ clearance activities can be therapeutically enhanced by modulating the TREM2/SIRPβ1/TYROBP signaling complex of the microglia. Given that GW5074 is neuroprotective and blood-brain barrier permeable, the identified drug GW5074 is of interest potentiality from both neuronal and microglial perspectives—killing two birds with one stone.

## Data Availability Statement

The original contributions presented in the study are included in the article/Supplementary Material, further inquiries can be directed to the corresponding author/s.

## Ethics Statement

The studies involving human participants were reviewed and approved by the Partners Human Research Committee, Columbia Human Research Protection Office Institutional Review Board of Rush University Medical Center. The patients/participants provided their written informed consent to participate in this study.

## Author Contributions

JB, WE, and EB designed and implemented the study. MR, SC, and EB wrote the manuscript. SC, MR, and KR conducted the experiments with technical assistance from KP and JS. DB coordinated the collection of blood, data from the memory, aging study, and contributed to the design of the study. All authors read and edited the manuscript.

## Conflict of Interest

EB and WE were founders of IMAD Therapeutics. The remaining authors declare that the research was conducted in the absence of any commercial or financial relationships that could be construed as a potential conflict of interest.

## Publisher’s Note

All claims expressed in this article are solely those of the authors and do not necessarily represent those of their affiliated organizations, or those of the publisher, the editors and the reviewers. Any product that may be evaluated in this article, or claim that may be made by its manufacturer, is not guaranteed or endorsed by the publisher.

## References

[B2] Avagyan H. Goldenson B. Tse E. Masoumi A. Porter V. Wiedau-Pazos M. (2009). Immune blood biomarkers of Alzheimer disease patients. *J. Neuroimmunol.* 210 67–72. 10.1016/j.jneuroim.2009.02.015 19329192

[B3] Barenholtz Levy H. (2021). Accelerated approval of aducanumab: Where do we stand now? *Ann. Pharmacother.* 56 736–739. 10.1177/10600280211050405 34595939

[B4] Bennett D. A. Buchman A. S. Boyle P. A. Barnes L. L. Wilson R. S. Schneider J. A. J. J. O. A. S. D. (2018). Religious orders study and rush memory and aging project. *J. Alzheimers Dis.* 64 S161–S189. 29865057 10.3233/JAD-179939 PMC6380522

[B5] Bennett D. A. Schneider J. A. Buchman A. S. Barnes L. L. Boyle P. A. Wilson R. S. (2012). Overview and findings from the rush memory and aging project. *Curr. Alzheimer Res.* 9 646–663. 10.2174/156720512801322663 22471867 PMC3439198

[B6] Bis J. C. Jian X. Kunkle B. W. Chen Y. Hamilton-Nelson K. L. Bush W. S. (2020). Whole exome sequencing study identifies novel rare and common Alzheimer’s-Associated variants involved in immune response and transcriptional regulation. *Mol. Psychiatry* 25 1859–1875. 30108311 10.1038/s41380-018-0112-7 PMC6375806

[B7] Bradshaw E. M. Chibnik L. B. Keenan B. T. Ottoboni L. Raj T. Tang A. (2013). CD33 Alzheimer’s disease locus: altered monocyte function and amyloid biology. *Nat. Neurosci.* 16 848–850. 10.1038/nn.3435 23708142 PMC3703870

[B8] Burgess S. Echeverria V. (2010). Raf inhibitors as therapeutic agents against neurodegenerative diseases. *CNS Neurol. Disord. Drug Targets* 9 120–127. 10.2174/187152710790966632 20201822

[B9] Chatila Z. K. Bradshaw E. M. (2021). Alzheimer’s disease genetics: a dampened microglial response? *Neuroscientist* [Online ahead of print]. 10.1177/10738584211024531 34142603

[B10] Chen H. M. Wang L. D’MELLO S. R. (2008). Inhibition of ATF-3 expression by B-Raf mediates the neuroprotective action of GW5074. *J. Neurochem.* 105 1300–1312. 10.1111/j.1471-4159.2008.05226.x 18194435

[B11] Chin P. C. Liu L. Morrison B. E. Siddiq A. Ratan R. R. Bottiglieri T. (2004). The c-Raf inhibitor GW5074 provides neuroprotection in vitro and in an animal model of neurodegeneration through a MEK-ERK and Akt-independent mechanism. *J. Neurochem.* 90 595–608. 10.1111/j.1471-4159.2004.02530.x 15255937

[B12] Claes C. Van Den Daele J. Boon R. Schouteden S. Colombo A. Monasor L. S. (2019). Human stem cell-derived monocytes and microglia-like cells reveal impaired amyloid plaque clearance upon heterozygous or homozygous loss of TREM2. *Alzheimers Dement.* 15 453–464. 10.1016/j.jalz.2018.09.006 30442540

[B13] Dahlgren K. N. Manelli A. M. Stine W. B. Jr. Baker L. K. Krafft G. A. Ladu M. J. (2002). Oligomeric and fibrillar species of amyloid-beta peptides differentially affect neuronal viability. *J. Biol. Chem.* 277 32046–32053. 10.1074/jbc.M201750200 12058030

[B14] Dhillon A. S. Hagan S. Rath O. Kolch W. (2007). MAP kinase signalling pathways in cancer. *Oncogene* 26 3279–3290. 10.1038/sj.onc.1210421 17496922

[B15] Echeverria V. Burgess S. Gamble-George J. Arendash G. W. Citron B. A. (2008). Raf inhibition protects cortical cells against beta-amyloid toxicity. *Neurosci. Lett.* 444 92–96. 10.1016/j.neulet.2008.07.092 18706973

[B16] Echeverria V. Ducatenzeiler A. Dowd E. Janne J. Grant S. M. Szyf M. (2004). Altered mitogen-activated protein kinase signaling, tau hyperphosphorylation and mild spatial learning dysfunction in transgenic rats expressing the beta-amyloid peptide intracellularly in hippocampal and cortical neurons. *Neuroscience* 129 583–592. 10.1016/j.neuroscience.2004.07.036 15541880

[B17] Ehrenreiter K. Piazzolla D. Velamoor V. Sobczak I. Small J. V. Takeda J. (2005). Raf-1 regulates Rho signaling and cell migration. *J. Cell Biol.* 168 955–964. 10.1083/jcb.200409162 15753127 PMC2171799

[B18] FDA (2021). *FDA Grants Accelerated Approval for Alzheimer’s Drug.* Available online at: https://www.fda.gov/news-events/press-announcements/fda-grants-accelerated-approval-alzheimers-drug

[B19] Fiala M. Lin J. Ringman J. Kermani-Arab V. Tsao G. Patel A. (2005). Ineffective phagocytosis of amyloid-beta by macrophages of Alzheimer’s disease patients. *J. Alzheimers Dis.* 7 221–232; discussion 255–262. 10.3233/jad-2005-7304 16006665

[B20] Gaikwad S. Larionov S. Wang Y. Dannenberg H. Matozaki T. Monsonego A. (2009). Signal regulatory protein-beta1: a microglial modulator of phagocytosis in Alzheimer’s disease. *Am. J. Pathol.* 175 2528–2539. 10.2353/ajpath.2009.090147 19893026 PMC2789620

[B21] Galabova-Kovacs G. Matzen D. Piazzolla D. Meissl K. Plyushch T. Chen A. P. (2006). Essential role of B-Raf in ERK activation during extraembryonic development. *Proc. Natl. Acad. Sci. U. S. A.* 103 1325–1330. 10.1073/pnas.0507399103 16432225 PMC1360532

[B22] Gosselin D. Skola D. Coufal N. G. Holtman I. R. Schlachetzki J. C. M. Sajti E. (2017). An environment-dependent transcriptional network specifies human microglia identity. *Science* 356:eaal3222. 10.1126/science.aal3222 28546318 PMC5858585

[B23] Guerreiro R. Wojtas A. Bras J. Carrasquillo M. Rogaeva E. Majounie E. (2013). TREM2 variants in Alzheimer’s disease. *N. Engl. J. Med.* 368 117–127. 23150934 10.1056/NEJMoa1211851 PMC3631573

[B24] Harold D. Abraham R. Hollingworth P. Sims R. Gerrish A. Hamshere M. L. (2009). Genome-wide association study identifies variants at CLU and PICALM associated with Alzheimer’s disease. *Nat. Genet.* 41 1088–1093. 10.1038/ng.440 19734902 PMC2845877

[B25] Haure-Mirande J. V. Wang M. Audrain M. Fanutza T. Kim S. H. Heja S. (2019). Integrative approach to sporadic Alzheimer’s disease: deficiency of TYROBP in cerebral Abeta amyloidosis mouse normalizes clinical phenotype and complement subnetwork molecular pathology without reducing Abeta burden. *Mol. Psychiatry* 24 431–446. 10.1038/s41380-018-0255-6 30283032 PMC6494440

[B26] Hollingworth P. Harold D. Sims R. Gerrish A. Lambert J. C. Carrasquillo M. M. (2011). Common variants at ABCA7, MS4A6A/MS4A4E, EPHA1, CD33 and CD2AP are associated with Alzheimer’s disease. *Nat. Genet.* 43 429–435. 10.1038/ng.803 21460840 PMC3084173

[B27] Jin S. C. Benitez B. A. Karch C. M. Cooper B. Skorupa T. Carrell D. (2014). Coding variants in TREM2 increase risk for Alzheimer’s disease. *Hum. Mol. Genet.* 23 5838–5846. 10.1093/hmg/ddu277 24899047 PMC4189899

[B28] Jonsson T. Stefansson H. Steinberg S. Jonsdottir I. Jonsson P. V. Snaedal J. (2013). Variant of TREM2 associated with the risk of Alzheimer’s disease. *N. Engl. J. Med.* 368 107–116. 23150908 10.1056/NEJMoa1211103 PMC3677583

[B29] Kim S. M. Mun B. R. Lee S. J. Joh Y. Lee H. Y. Ji K. Y. (2017). TREM2 promotes Abeta phagocytosis by upregulating C/EBPalpha-dependent CD36 expression in microglia. *Sci. Rep.* 7:11118. 10.1038/s41598-017-11634-x 28894284 PMC5593901

[B30] Kleinberger G. Yamanishi Y. Suarez-Calvet M. Czirr E. Lohmann E. Cuyvers E. (2014). TREM2 mutations implicated in neurodegeneration impair cell surface transport and phagocytosis. *Sci. Transl. Med.* 6:243ra86. 10.1126/scitranslmed.3009093 24990881

[B31] Lambert J. C. Heath S. Even G. Campion D. Sleegers K. Hiltunen M. (2009). Genome-wide association study identifies variants at CLU and CR1 associated with Alzheimer’s disease. *Nat. Genet.* 41 1094–1099. 10.1038/ng.439 19734903

[B32] Lambert J. C. Ibrahim-Verbaas C. A. Harold D. Naj A. C. Sims R. Bellenguez C. (2013). Meta-analysis of 74,046 individuals identifies 11 new susceptibility loci for Alzheimer’s disease. *Nat. Genet.* 45 1452–1458. 10.1038/ng.2802 24162737 PMC3896259

[B33] Lee C. Y. Landreth G. E. (2010). The role of microglia in amyloid clearance from the AD brain. *J. Neural Transm.* 117 949–960. 10.1007/s00702-010-0433-4 20552234 PMC3653296

[B34] Mei M. Su B. Harrison K. Chao M. Siedlak S. L. Previll L. A. (2006). Distribution, levels and phosphorylation of Raf-1 in Alzheimer’s disease. *J. Neurochem.* 99 1377–1388. 10.1111/j.1471-4159.2006.04174.x 17064357

[B35] Mercer K. Chiloeches A. Huser M. Kiernan M. Marais R. Pritchard C. (2002). ERK signalling and oncogene transformation are not impaired in cells lacking A-Raf. *Oncogene* 21 347–355. 10.1038/sj.onc.1205101 11821947

[B36] Mikula M. Schreiber M. Husak Z. Kucerova L. Ruth J. Wieser R. (2001). Embryonic lethality and fetal liver apoptosis in mice lacking the c-raf-1 gene. *EMBO J.* 20 1952–1962. 10.1093/emboj/20.8.1952 11296228 PMC125416

[B37] Mullard A. (2021). Landmark Alzheimer’s drug approval confounds research community. *Nature* 594 309–310. 10.1038/d41586-021-01546-2 34103732

[B38] Naj A. C. Jun G. Beecham G. W. Wang L. S. Vardarajan B. N. Buros J. (2011). Common variants at MS4A4/MS4A6E, CD2AP, CD33 and EPHA1 are associated with late-onset Alzheimer’s disease. *Nat. Genet.* 43 436–441. 10.1038/ng.801 21460841 PMC3090745

[B39] O’NEILL E. Rushworth L. Baccarini M. Kolch W. (2004). Role of the kinase MST2 in suppression of apoptosis by the proto-oncogene product Raf-1. *Science* 306 2267–2270. 10.1126/science.1103233 15618521

[B40] Pearson G. Robinson F. Beers Gibson T. Xu B. E. Karandikar M. Berman K. (2001). Mitogen-activated protein (MAP) kinase pathways: regulation and physiological functions. *Endocr. Rev.* 22 153–183. 10.1210/edrv.22.2.0428 11294822

[B41] Peng Q. Malhotra S. Torchia J. A. Kerr W. G. Coggeshall K. M. Humphrey M. B. (2010). TREM2- and DAP12-dependent activation of PI3K requires DAP10 and is inhibited by SHIP1. *Sci. Signal.* 3:ra38. 10.1126/scisignal.2000500 20484116 PMC2900152

[B42] Robinson M. J. Cobb M. H. (1997). Mitogen-activated protein kinase pathways. *Curr. Opin. Cell Biol.* 9 180–186. 9069255 10.1016/s0955-0674(97)80061-0

[B43] Ryan K. J. White C. C. Patel K. Xu J. Olah M. Replogle J. M. (2017). A human microglia-like cellular model for assessing the effects of neurodegenerative disease gene variants. *Sci. Transl. Med.* 9:eaai7635. 10.1126/scitranslmed.aai7635 29263232 PMC5945290

[B44] Seshadri S. Fitzpatrick A. L. Ikram M. A. Destefano A. L. Gudnason V. Boada M. (2010). Genome-wide analysis of genetic loci associated with Alzheimer disease. *JAMA* 303 1832–1840. 10.1001/jama.2010.574 20460622 PMC2989531

[B45] Takahashi K. Rochford C. D. Neumann H. (2005). Clearance of apoptotic neurons without inflammation by microglial triggering receptor expressed on myeloid cells-2. *J. Exp. Med.* 201 647–657. 10.1084/jem.20041611 15728241 PMC2213053

[B46] Varga A. Ehrenreiter K. Aschenbrenner B. Kocieniewski P. Kochanczyk M. Lipniacki T. (2017). RAF1/BRAF dimerization integrates the signal from RAS to ERK and ROKalpha. *Sci. Signal.* 10:eaai8482. 10.1126/scisignal.aai8482 28270557

[B47] Walsh D. M. Klyubin I. Fadeeva J. V. Cullen W. K. Anwyl R. Wolfe M. S. (2002). Naturally secreted oligomers of amyloid beta protein potently inhibit hippocampal long-term potentiation in vivo. *Nature* 416 535–539. 10.1038/416535a 11932745

[B48] Wildsmith K. R. Holley M. Savage J. C. Skerrett R. Landreth G. E. (2013). Evidence for impaired amyloid beta clearance in Alzheimer’s disease. *Alzheimers Res. Ther.* 5:33. 10.1186/alzrt187 23849219 PMC3978761

[B49] Wojnowski L. Stancato L. F. Zimmer A. M. Hahn H. Beck T. W. Larner A. C. (1998). Craf-1 protein kinase is essential for mouse development. *Mech. Dev.* 76 141–149. 10.1016/s0925-4773(98)00111-7 9767153

[B50] Yamaguchi O. Watanabe T. Nishida K. Kashiwase K. Higuchi Y. Takeda T. (2004). Cardiac-specific disruption of the c-raf-1 gene induces cardiac dysfunction and apoptosis. *J. Clin. Invest.* 114 937–943. 10.1172/JCI20317 15467832 PMC518660

[B51] Yao H. Coppola K. Schweig J. E. Crawford F. Mullan M. Paris D. (2019). Distinct signaling pathways regulate TREM2 phagocytic and NFkappaB antagonistic activities. *Front. Cell Neurosci* 13:457. 10.3389/fncel.2019.00457 31649511 PMC6795686

[B52] Zaghi J. Goldenson B. Inayathullah M. Lossinsky A. S. Masoumi A. Avagyan H. (2009). Alzheimer disease macrophages shuttle amyloid-beta from neurons to vessels, contributing to amyloid angiopathy. *Acta Neuropathol.* 117 111–124. 10.1007/s00401-008-0481-0 19139910 PMC5650912

