# Supplementary material for: GW5074 Increases Microglial Phagocytic Activities: Potential Therapeutic Direction for Alzheimer’s Disease
Source: Front Cell Neurosci. 2022 May 23;16:894601. doi: 10.3389/fncel.2022.894601 (PMC9169965; doi:10.3389/fncel.2022.894601)
Supplement: Supplementary file 1 [file Data_Sheet_1.docx]

**Supplementary Material**


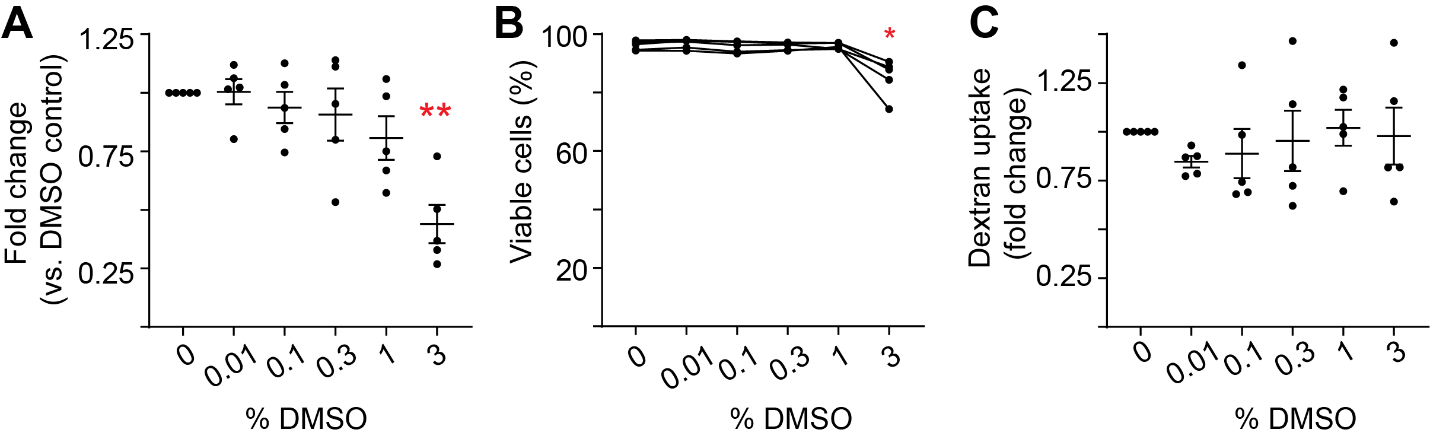


**Supplementary Figure 1:DMSO tolerance test.** Monocytes were seeded into a 384 well plate and differentiated to MDMi. MDMi were subjected to a titration of DMSO with 0%, 0.01%, 0.1%, 0.3% 1% and 3% DMSO. On day 10, cells were incubated with FITC-labeled dextran for 2 hours and labeled with a live dead cell stain before being imaged on the IXM-C. **A)** Cell number depending on DMSO concentration at different concentrations. **B)** Cell viability depending on DMSO concentration at different concentrations. **C)** Dextran signal intensity depending on DMSO concentration at different concentrations. One-way ANOVA with Dunnett's multiple comparisons test. *p<0.05; **p<0.01.

**
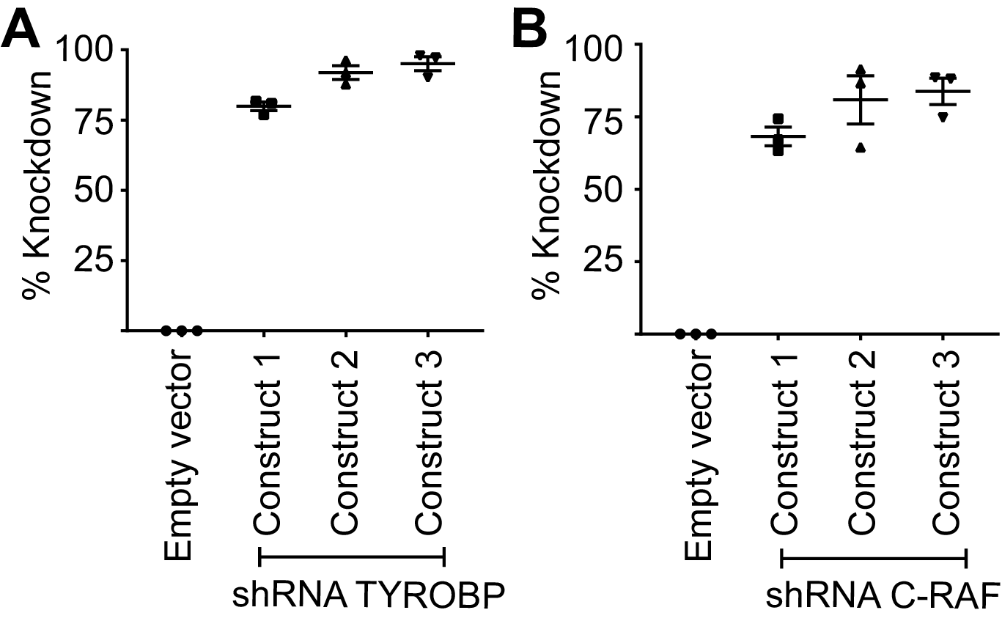
**

**Supplementary Figure 2: TYROBP and C-RAF knockdown in MDMi. A)** Lentiviral mediated shRNA knockdown of TYROBP in MDMi. Three shRNA constructs were used to knockdown TYROBP. **B)** Lentiviral mediated shRNA knockdown of c-RAF in MDMi. A total of three constructs were used.

**Supplementary Table 1**: **Drug screen “hits” from the LOPAC and their functions***

| **Compound Name** | **Class** | **Enzyme** | **Action** | **Selectivity** | **Description** |
| --- | --- | --- | --- | --- | --- |
| Acetylsalicylic acid | Prostaglandin | Enzyme | Inhibitor | COX-3 > COX-1 > COX-2 | COX inhibitor; antithrombotic |
| 5,7-Dichlorokynurenic acid | Glutamate |  | Antagonist | NMDA-glycine | Potent excitatory amino acid receptor antagonist; active at the strychnine-insensitive glycine binding site of the NMDA glutamate receptor |
| GW5074 | Phosphorylation | Enzyme | Inhibitor | Raf1 kinase | cRaf1 kinase inhibitor |
| Ro 90-7501 | Neurodegeneration |  | Inhibitor | amyloid beta42 fibril formation | Inhibits amyloid beta42 (Abeta42) fibril formation. |
| SMER28 | Cell Biology |  | Enhancer | Autophagy | Small molecule modulator of mammalian autophagy. |
| Retinoic acid | Apoptosis | Enzyme | Activator |  | Induces caspase-dependent apoptosis |
| Tocainide hydrochloride | Na+ Channel |  | Blocker |  | Sodium channel blocker. Class IB antiarrhythmic. |
| Diethylenetriaminepentaacetic acid | Biochemistry | Enzyme | Inhibitor | Zn2+-dependent  protease | Zinc-dependent proteases carboxypeptidase A andaminopeptidase N inhibitor; chelating agent |
| Dihydroouabain | Ion Pump |  | Inhibitor | Na+/K+ Pump | Sodium-potassium pump inhibitor |
| SCH-28080 | Ion Channels | Enzyme | Inhibitor | H+/K+-ATPase | Potent inhibitor of gastric H+ and K+-ATPase. |
| 1-Deoxynojirimycin hydrochloride | Biochemistry | Enzyme | Inhibitor | alpha-glucosidase | alpha-glucosidase I-II inhibitor |
| Dobutamine hydrochloride | Adrenoceptor |  | Agonist | beta1 | beta1 Adrenoceptor agonist |
| Dilazep hydrochloride | Adenosine |  | Inhibitor | Uptake | Adenosine uptake inhibitor |
| Efaroxan hydrochloride | Imidazoline |  | Antagonist | I1 | I1 Imidazoline binding site antagonist |
| Ganaxolone | GABA |  | Modulator | GABA-A | Positive allosteric modulator of GABA-A receptors |
| Desipramine hydrochloride | Adrenoceptor |  | Inhibitor | Uptake | Antidepressant |
| Idarubicin | DNA Metabolism |  | Inhibitor |  | Antineoplastic |
| GW2974 | Phosphorylation | Enzyme | Inhibitor | EGFR / ErbB-2 | Dual EGFR and ErbB-2 receptor tyrosine kinase inhibitor |
| Ellipticine | Cell Cycle | Enzyme | Inhibitor | CYP1A1 / TopoII | Cytochrome P450 (CYP1A1)and DNA topoisomerase II inhibitor |
| m-Iodobenzylguanidinehemisulfate | Apoptosis |  | Activator |  | Antitumor agent which inhibits ADP ribosylation; induces  changes in the mitochondrial membrane potential,  activation of caspase-3 and DNA fragmentation |
| (±)-alpha-Methyl-4-carboxyphenylglycine | Glutamate |  | Antagonist | Metabotropic | Competitive metabotropic glutamate receptor antagonist |
| Olvanil | Neurotransmission |  | Agonist | Vanilloidreceptor | Vanilloid receptor agonist |
| Topotecan hydrochloride hydrate | Apoptosis and Cell  Cycle | Enzyme | Inhibitor | topoisomerase I | Topetecan is a topoisomerase I inhibitor and an apoptosisinducer. It is a potent antineoplastic agent |
| Pirfenidone | Immune System |  | Inhibitor |  | Inhibitor of collagen production and fibroblastproliferation |
| Moxonidine hydrochloride | Adrenoceptor |  | Agonist | alpha2A | Selective alpha2A adrenoreceptor agonist; imidazoline  binding site agonist; antihypertensive |
| S-Methylisothioureahemisulfate | Nitric Oxide | Enzyme | Inhibitor | iNOS | Selective inducle nitric oxide synthase inhibitor |
| Quercetin dihydrate | Cyclic Nucleotides | Enzyme | Inhibitor | PDE | Mitochondrial ATPase and phosphodiesterase (PDE)inhibitor; inhibits PI3-K activity |
| Gossypol | Apoptosis |  | Inducer |  | Natural product from cotton seeds with a variety of cell biological activities.  Proapoptotic, antimalarial, PKCinhibition. |
| Phenylephrine hydrochloride | Adrenoceptor |  | Agonist | alpha1 | alpha1 Adrenoceptor agonist; mydriatic; decongestant |
| alpha,beta-Methylene adenosine 5'-triphosphate  dilithium | P2 Receptor |  | Agonist |  | P2X > P2Y |
| Ivermectin | Cholinergic |  | Modulator | alpha7 nACh | Positive allosteric modulator of alpha7 neuronal nicotinic  acetylcholine receptor; also modulates glutamate-GABAactivatedchloride channels |
| K 185 | Melatonin |  | Antagonist |  | Melatonin receptor antagonist |
| PNU-282987 | Cholinergic |  | Agonist | Nicotinic alpha7 | Selective alpha7 nicotinic acetylcholine receptor (nAChR)  agonist. |
| ODQ | Cyclic Nucleotides | Enzyme | Inhibitor | NO-sensitive guanylyl  cyclase | Potent and selective NO-sensitive guanylyl cyclaseinhibitor |
| Lidocaine hydrochloride | Na+ Channel |  | Modulator |  | Anti-arrhythmic; local anesthetic |
| Ketorolac tris salt | Prostaglandin | Enzyme | Inhibitor | COX | Non-steroidal anti-inflammatory (NSAID) drug |
| loxoprofen | Prostaglandin | Enzyme | Inhibitor | COX | Non-steroidal anti-inflammatory (NSAID) drug; analgesic |
| Lomefloxacin hydrochloride | Antibiotic | Enzyme | Inhibitor | DNA Gyrase | DNA gyrase inhibitor |
| Loratadine | Histamine |  | Antagonist | H1 | H1 Histamine receptor antagonist |
| alpha-Lobeline hydrochloride | Cholinergic |  | Agonist | Nicotinic | Neuronal nicotinic acetylcholine receptor agonist |
| L-655,708 | Benzodiazepine |  | Ligand | GABA-A | Selective ligand for the benzodiazepine site of GABA-A receptors which contain the alpha5 subunit |
| Loxapine succinate | Dopamine |  | Antagonist |  | Dibenzoxazepine antipsychotic agent |
| Cysteamine hydrochloride | Somatostatin |  | Depleter |  | Induces duodenal ulcers in rats, potent depletion of prolactinconcentrations in pituitary tissue in vivo and in vitro and  depletion of somatostatin concentrations. |
| NG-Monomethyl-L-arginine acetate | Nitric Oxide | Enzyme | Inhibitor | NOS | Nitric oxide synthase inhibitor; blocks formation of endothelium-derived relaxing factor (EDRF) |
| 1-Methylhistamine dihydrochloride | Histamine |  | Metabolite |  | Histamine metabolite that crosses the blood-brain barrier |
| N6-Methyladenosine | Adenosine |  | Agonist |  | Adenosine analog useful for an adenosine reference standard since itis a poor substrate for adenosine deaminase |
| MK-912 | Adrenoceptor |  | Agonist | alpha2A | Selective alpha2A adrenoreceptor agonist |
| MK-886 | Leukotriene |  | Inhibitor |  | Potent and specific inhibitor of leukotriene biosynthesis |
| S-Methyl-L-thiocitrulline acetate | Nitric Oxide | Enzyme | Inhibitor | NOS | Potent inhibitor of NOS; more potent than L-thiocitrulline |
| (±)-Methoxyverapamil hydrochloride | Ca2+ Channel |  | Antagonist | L-type | L-type Ca2+ channel antagonist |
| MRS 2159 | P2 Receptor |  | Antagonist | P2X1 | P2X1 receptor antagonist |
| SB 216763 | Phosphorylation | Enzyme | Inhibitor | GSK-3 | Potent, selective, cell permeable inhibitor of glycogen synthetase  kinase-3 (GSK-3). |
| Quinelorane dihydrochloride | Dopamine |  | Agonist | D2 | D2 Dopamine receptor agonist; 2-aminopyridine analog ofQuinpirole |
| Ro 41-0960 | Neurotransmission | Enzyme | Inhibitor | COMT | Specific, reversible, orally-active COMT-inhibitor |
| Lidocaine N-ethyl bromide quaternary salt | Na+ Channel |  | Antagonist |  | Quaternary lidocaine derivative which blocks both fast,Na+-dependent action potentials and voltage-dependent,  non-inactivating Na+ conductance |
| SU 6656 | Phosphorylation | Enzyme | Inhibitor | Src family kinase | Selective Src family kinase inhibitor. |
| Spironolactone | Hormone |  | Antagonist | Mineralocorticoid | Competitive antagonist of the cytoplasmic aldosteronereceptor; diuretic |
| S(-)-3PPP hydrochloride | Dopamine |  | Agonist | D2 | Dopamine autoreceptor agonist; postsynaptic dopaminereceptor antagonist |
| SID7969543 | Gene Regulation | Enzyme | Inhibitor | SF-1 | A SF-1 (or NR5A1) inhibitor. |
| Trifluoperazine dihydrochloride | Dopamine |  | Antagonist | D1/D2 | Calmodulinantagonist; dopamine receptor antagonist;  antipsychotic; sedative |
| N-p-Tosyl-L-phenylalanine chloromethyl ketone | Biochemistry | Enzyme | Inhibitor | Chymotrypsin alpha | Blocks LPS- or cytokine-induced activation of nuclear  factor kB (NFkB), which, blocks the induction of iNOS andCOX-2 transcription; blocks activation of pp70s6k by allmitogens |
| Sulfaphenazole | Multi-Drug  Resistance | Enzyme | Inhibitor | Cytochrome P4502C | Antibacterial; inhibitor of cytochrome P4502C enzymes |
| BMS-193885 | Neurotransmission |  | Antagonist | Y1 | BMS-193885 is a potent, selective Y1 antagonist that is  active in both acute and chronic animal models of food  intake |
| SC 19220 | Prostaglandin |  | Antagonist | EP1 | EP1 Prostanoid receptor antagonist |
| Bay 11-7082 | Phosphorylation |  | Inhibitor | IKB-alpha | Inhibitor of cytokine-induced IKB-alpha phosphorylation |
| Sanguinarine chloride | Ion Pump |  | Inhibitor | Na+/K+ ATPase | Inhibitor of Mg2+ and Na+/K+-ATPase; isolated from the  leaves and stems of Macleaya cordata and microcarpa |
| SKF 96365 | Ca2+ Channel |  | Inhibitor |  | Selective inhibitor of receptor-mediated and voltage-gated  Ca2+ entry |
| SC-57461A | Lipids | Enzyme | Inhibitor | leukotriene A4 hydrolase | SC-57461A is an inhibitor ofleukotriene A4 hydrolase |
| (-)-Quinpirole hydrochloride | Dopamine |  | Agonist | D2/D3 | Selective D2-like dopamine receptor agonist with some selectivity for D3 sites |
| BRL 52537 hydrochloride | Neurotransmission |  | Agonist | kappa/mu opioid | Kappa/mu opioid receptor agonist. |
| Phenylbutazone | Prostaglandin | Enzyme | Substrate | Prostaglandin peroxidase | Anti-inflammatory; substrate for prostaglandin peroxidase |
| Phenamilmethanesulfonate | Na+ Channel |  | Inhibitor | Amiloride sensitive | Irreversible inhibitor of amiloride-sensitive Na+ channels; derivative of amiloride |
| Spiperone hydrochloride | Dopamine |  | Antagonist | D2 | Selective D2 dopamine receptor antagonist. |
| Spermine tetrahydrochloride | Glutamate |  | Antagonist | NMDA-Polyamine | Binds to the polyamine modulatory site of the NMDA glutamate receptor, attenuating both NMDA and quisqualatemediated responses in vivo. |
| SCH-202676 hydrobromide | G protein |  | Modulator | GPCR | Allosteric agonist and antagonist of G protein coupled receptors (GPCRs) |
| SR 2640 | Leukotriene |  | Antagonist | CysLT1 | Subtype specific CysLT1 leukotriene receptor antagonist. |
| Albuterol hemisulfate | Adrenoceptor |  | Agonist | beta2 | beta Adrenoceptor agonist |
|  | Ca2+ Channel |  | Inhibitor |  | Selective inhibitor of receptor-mediated and voltage-gated Ca2+ entry |
| Salmeterol xinafoate | Adrenoceptor |  | Agonist | beta2 | beta2 Adrenoceptor agonist |
| SU 5416 | Phosphorylation |  |  | VEGFR PTK | Potent and selective VEGFR PTK inhibitor; inhibits VEGF- induced  angiogenesis |
| (+)-Quisqualic acid | Glutamate |  | Agonist | AMPA | Active enantiomer of quisqualic acid; excitatory amino acid at glutamatereceptors; anthelmentic agent |
| LE 300 | Dopamine |  | Antagonist | D1 | Potent, selective D1 dopamine receptor antagonist |
